# Oxytocin Mitigates VEGF Expression in Differentiated RPE cells – A Novel Therapeutic Approach for Diabetic Retinopathy

**Published:** 2025-12-08

**Authors:** Daira Yepez, Cristian Mercado, Gabriel Ordonez, Laura Valdez, Khalid Benamar, Andrew Tsin

**Affiliations:** 1 Department of Neuroscience, The University of Texas Rio Grande Valley School of Medicine, Edinburg, Texas, USA; 2 Paul L Foster School of Medicine, Texas Tech University Health Science Center, El Paso, Texas, USA

**Keywords:** oxytocin, hyperglycemia, diabetic retinopathy, VEGF, retinal pigment epithelium, ARPE-19, neovascularization

## Abstract

Diabetic retinopathy (DR) is a leading cause of vision loss, driven largely by vascular endothelial growth factor (VEGF)-induced neovascularization and vascular permeability. Current therapies rely on intravitreal anti-VEGF injections that mitigate VEGF action, but they require repeated administration, can cause complications, and are ineffective in some patients. Oxytocin (OXT), a neuropeptide best known for reproductive and social roles, has recently been implicated in metabolic regulation and vascular signaling. Evidence from animal models suggests that OXT may reduce VEGF expression in the retina. However, the underlying molecular mechanism is not known. The present study seeks to investigate the cellular and molecular bases of this VEGF inhibition by OXT. Results from our experiments revealed that OXT significantly reduced VEGF secretion by ARPE-19 cells in a dose- and time-dependent manner. Together with in-vivo studies on OXT and VEGF reduction in animal models, our results support the suggestion that OXT is a candidate for upstream therapeutic intervention of DR at an early stage of disease development.

## Introduction

Diabetic retinopathy (DR) is a common complication of diabetes and a leading cause of vision impairment worldwide [1]. It progresses from non-proliferative DR, marked by microaneurysms and macular edema, to proliferative DR, where pathological neovascularization and hemorrhaging threaten vision. A central driver of these changes is the overexpression of vascular endothelial growth factor (VEGF) in response to hypoxia caused by chronic hyperglycemia. Elevated VEGF stimulates angiogenesis and vascular permeability, disrupting the blood–retina barrier and accelerating vision loss [2,3].

VEGF is a family of proteins, the most relevant of which is VEGF-A encoded by the VEGFA gene on chromosome 6p21.1. The most common isoform secreted by RPE cells is VEGF121 and VEGF165 [4,5]. VEGF-A normally triggers cell action by binding to VEGF receptor-1 and VEGF receptor-2, which are tyrosine kinase receptors on the outer cell membrane. Activation of VEGFR2 triggers angiogenesis [6] while VEGFR1 binds to excess VEGF. This is also the aim of anti-VEGF therapies: inhibitors bind to VEGF to prevent it from activating VEGFR2. With few exceptions, VEGF inhibitors are antibodies that bind to VEGF-A to prevent growth of new blood vessels, making them valuable treatments for various cancers as well as DR.

While anti-VEGF injections effectively reduce DR disease progression, they require frequent intravitreal delivery, are costly, and fail in 10–50% of patients [7–10]. Other therapies such as laser photocoagulation or vitrectomy remain invasive and do not target underlying molecular drivers.

The retinal pigment epithelium (RPE) is made up of highly specialized polarized epithelial cells that provide an interface between photoreceptors in the retina and the choroid. It plays a central role in eye health by secreting VEGF to maintain proper circulation in the surrounding vasculature. In diabetes, however, RPE cells overproduce VEGF [11] due to oxidative stress and hypoxia-inducible factor (HIF) signaling [12,13]. ARPE-19, a human-derived RPE model, provides an in vitro system for studying VEGF secretion under stress conditions. Although less differentiated than native RPE, they retain VEGF regulatory pathways and are widely used in retinal research.

A recent study reported an effective method to induce ARPE-19 differentiation in culture by enriching culture media with nicotinamide, also highlighting the important role of metabolism in cell differentiation [14]. This approach provides an the opportunity to examine cellular and molecular properties of differentiated ARPE-19 in vitro, including VEGF production in response to exogenous glucose treatments.

Oxytocin (OXT) is a nonapeptide hormone, also known as the “love hormone”, classically associated with parturition, lactation, and social bonding. Beyond reproduction, it has been shown to regulate metabolism, inflammation, and vascular function [15–18]. OXT is concentrated in the central nervous system though it has a strong presence in the retina as well [19]. OXT receptors (OXTR) are a type of g-protein coupled receptor known to increase intracellular calcium and activate the PKC pathway when activated [20].

Recent animal studies suggest OXT treatment given by intraperitoneal or intravitreal injections reduces VEGF levels in diabetic retinas and plasma [21]. Given that OXT levels decrease in diabetes and obesity patients [22], OXT supplementation may provide a protective effect against DR development via an inhibition of VEGF expression. However, the cellular and molecular mechanism of this VEGF inhibition has not been investigated.

In the present study, we hypothesize that OXT inhibits VEGF secretion by its action on the oxytocin receptor in the retinal pigment epithelium (RPE) leading to an inhibition of VEGF synthesis and release by the RPE, a major producer of VEGF in the retina. Therefore, the present study aims to evaluate the effect of OXT on VEGF secretion in differentiated ARPE-19 cells cultured under low- and high-glucose conditions. Previous in-vivo studies suggest OXT decreased VEGF concentrations in plasma and VEGF protein expression in the retina [21]. Results from our cellular investigation have elucidated a cellular and molecular pathway for in-vivo report on the inhibition of VEGF by oxytocin. Therefore, the use of OXT as a therapeutic agent is a novel approach for DR intervention at an early stage of this disease development.

## Material and methods

ARPE-19 human retinal pigment epithelial cells (ATCC. Manassas, VA) were cultured in low (4.5 mM) or high (30 mM) glucose in MEM-F12 (non-differentiation) and in differentiation media: MEM-nic [14] in 6 well plates for at least a week. Cells were then treated with oxytocin (OXT, ThermoFisher Chemical Co., Ward Hill, MA) at low (1 pg/mL) or medium (100 pg/mL) concentrations. Media was collected at 24 and 72 hours for VEGF quantification by ELISA (R+D Systems, Minneapolis, Mn). Oxytocin receptor (OXTR) in the cell pellet was assayed by ELISA (MyBioSource, San Diego, CA). All photographs were taken with the same Nikon DS-Fi3 camera mounted on a Nikon Eclipse Ts2R microscope. GraphPad Prism 10 was used to analyze data and create graphs. Several sets of experiments were conducted using either non-differentiation or differentiation media. Results from experiments using differentiated ARPE-19 are reported in the Results section. Statistical analyses were performed using ANOVA with a 95\% confidence threshold.

## Results

**Figure 1.**
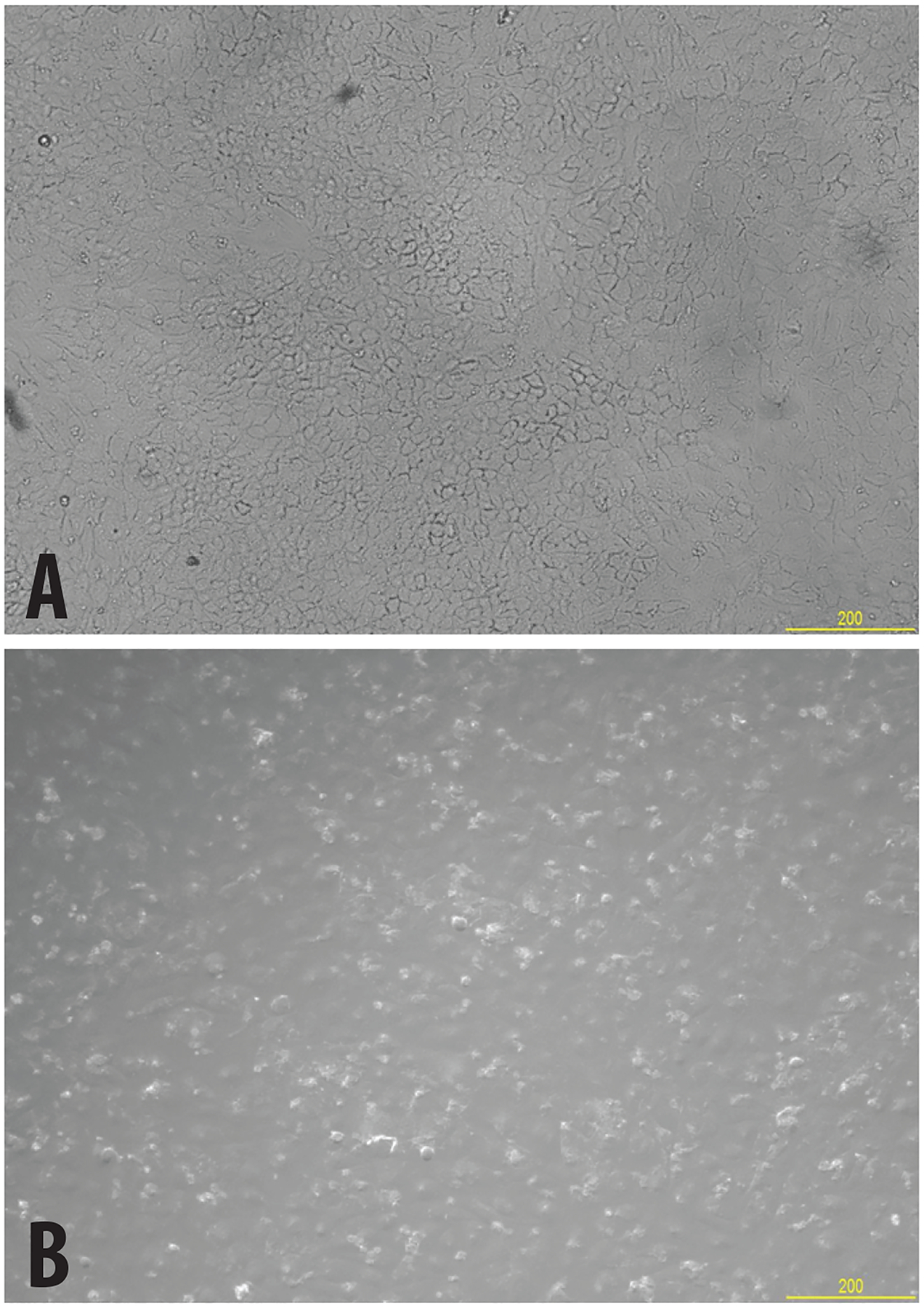
Photomicrograph of a) differentiated ARPE-19 and b) undifferentiated ARPE-19 cells cultured in MEM-nic. Scale in micrometers.

ARPE-19 cells were cultured for at least a week before treatments began, and cell differentiation was identified by visual examination (Figures 1a & b). No observable change in differentiation was noted between treated, untreated, high, or low glucose groups. OXT treatment maintained or slightly improved cell counts and viability, which is consistent with its potential cytoprotective role [21].

**Figure 2.**
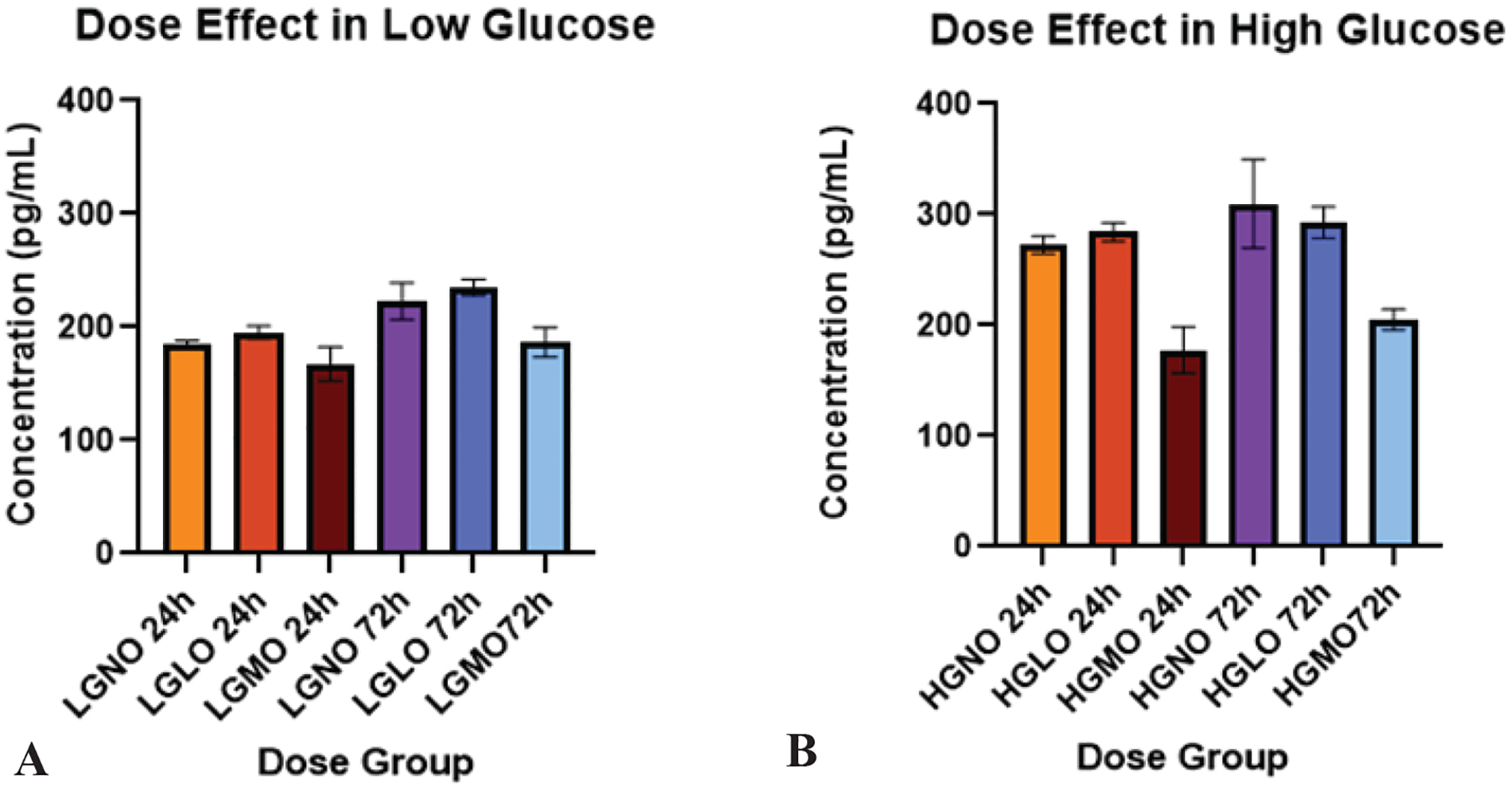
I**n**hibition of VEGF Expression by OXT in Differentiated ARPE-19. A) Effect of no, low, and medium doses of OXT on cells cultured in low glucose for 24 and 72 hours. b) Effect of no, low, and medium doses of OXT on cells cultured in high glucose for 24 and 72 hours. There was no significant dose effect on VEGF levels in normoglycemic ARPE cells treated with OXT for 24h and 72h. However, there was a significant increase in VEGF level from 24h to 72h in ARPE-19 cells with the lowest dose OXT treatment (i.e.. LGLO 24h vs LGLO 72h, Fig 2a). In comparison, there was a significant dose effect on VEGF levels in hyperglycemic ARPE-19 cells treated with OXT for 24 and 72 hours. The addition of OXT significantly reduced the VEGF expression by ARPE-19 cells in high glucose media (Fig 2b).

There was no significant dose effect on VEGF levels in normoglycemic ARPE-19 cells treated with OXT for 24h and 72h (Figure 2a; Table 1). In comparison, there was a significant dose effect on VEGF levels in hyperglycemic ARPE-19 cells treated with OXT for 24H and 72H. The addition of OXT significantly reduced the VEGF expression by ARPE-19 cells (Figure 2b, Table 1).

**Figure 3.**
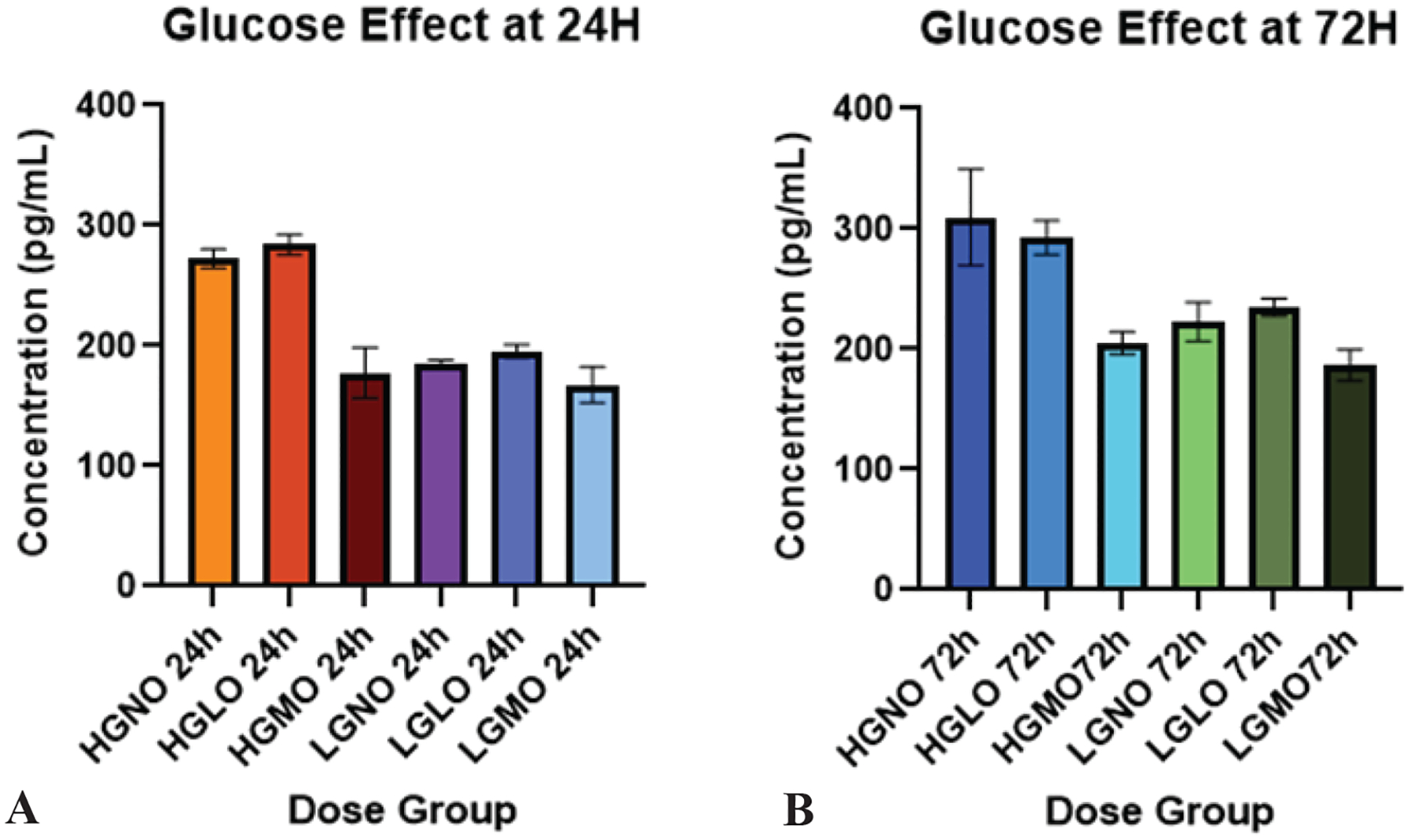
Inhibition of VEGF Expression by Low Glucose in Differentiated ARPE-19 cells. a) Effect of glucose concentration on cells cultured in no, low, and medium OXT doses after 24 hours. b) Effect of glucose concentration on cells cultured in no, low, and medium OXT doses after 72 hours. High glucose treatment resulted in a significantly higher level of VEGF at 24h (Fig 3a) and at 72h (Fig.3b) in all low dose treatment groups and at 24h in the no OXT group. This is consistent with hyperglycemic condition leading to higher VEGF expression. However, there is no significant difference between VEGF levels in ARPE-19 cells treated for 24h vs 72h with the medium OXT dose (Fig 3a vs Fig 3b).

High glucose treatment resulted in a significantly higher level of VEGF at 24h (Figure 3a) and at 72h (Figure3b) in all treatment groups. This is consistent with hyperglycemic condition leading to higher VEGF expression. However, there is no significant difference between VEGF levels in hyperglycemic ARPE-19 cells treated for 24h vs 72h, though there is a significant difference between VEGF levels in normoglycemic ARPE-19 cells treated for 24h vs 72h (Figure 3a vs Figure 3b).

## Discussion

This study examined the role of oxytocin (OXT) in the inhibition of VEGF expression in differentiated ARPE-19 cells under diabetic (high glucose) and normal (low glucose) conditions. High glucose is known to have a positive correlation with VEGF secretion in RPE cells, including the ARPE-19 cell line [23], and this trend is consistently observed in experiments in the present study (Fig 3 a and b). Hyperglycemia induces oxidative stress and hypoxia, mimicking diabetic conditions that the cells react to by secreting higher levels of VEGF when left untreated. Our results suggest that OXT has a statistically significant inhibitory effect on VEGF secretion, particularly under hyperglycemic stress.

Notably, VEGF concentrations in hyperglycemic media treated with OXT (add concentration of MO) were consistently lowered to those measured in normoglycemic groups (Fig 2b). This may suggest that OXT treatment normalized the VEGF secretion of cells cultured in hyperglycemic conditions. These findings support other earlier in vivo work [21] showing that OXT treatment reduced VEGF levels in diabetic rat retinas and plasma. Because OXT is not known to bind or interact with VEGF, it is likely to decrease VEGF protein expression rather than inhibiting VEGF, as in clinical anti-VEGF therapy. This is also supported by the above-mentioned in vivo experiment, which found a decrease in the expression of VEGF protein in OXT-treated rat retinas. To our knowledge, the present study is the first in vitro studies to show that differentiated ARPE-19 expressed less VEGF in response to exogenous OXT, thus providing a cellular/molecular pathway to substantiate results of animal model studies.

A key observation in the present study was the non-linear dose–response relationship. Treatment with OXT at the LO concentrations did not result in a significant inhibition while MO concentration of OXT lead to a significant inhibition of VEGF expression (Fig 2b). Detailed mechanism of this dose-dependent effect awaits further investigations.

Results in our previous experiments show that ARPE-19 cells express measurable OXTR and OXT treatment modulated OXTR expression in a dose-dependent manner. Our experiments also showed OXTR expression was significantly upregulated under high glucose, indicating that retinal pigment epithelial cells may become more sensitive to OXT during diabetic stress. This adaptive response could explain why OXT treatment had greater inhibitory and cytoprotective effects under high glucose conditions. The data also suggest that OXT may act upstream of VEGF secretion, potentially influencing transcriptional or post-translational regulation rather than secretion itself, possibility because retinal cells were more responsive to OXT under diabetic conditions.

OXT treatment appeared to improve cell viability, particularly under low glucose conditions. This aligns with reports on OXT’s cytoprotective and anti-apoptotic properties in other tissues. Although experimental variability limits firm conclusions, the trend suggests that OXT could preserve RPE cell health in addition to modulating VEGF.

Our results provide strong evidence in support of a cellular and molecular pathway whereby OXT inhibits VEGF expression in the retina of animal models. Further studies using primary RPE cells, as well as in vivo models, will provide additional details on the mechanism and application on therapeutics.

## Conclusion

This study provides evidence that oxytocin inhibits VEGF expression in differentiated human ARPE-19 cells, with effects most pronounced under high glucose conditions. OXT may act upstream of VEGF secretion, offering a novel approach to mitigate diabetic retinopathy. These results support the potential of OXT as a preventive or adjunctive therapy for diabetic retinopathy, warranting further investigation into dosing strategies, mechanisms of action, and translational studies in animal models and humans..

## Figures and Tables

**Table 1.** Statistical Significance of OXT Inhibition of VEGF Expression by ARPE-19 cells in hyperglycemia (HG).

One-way ANOVA P-values (Significant = P<0.05)	
All LG 24H	0.1799
All HG 24H	0.0031
All LG 72H	0.0816
All HG 72H	0.0521

## References

[1] Shukla UV, Tripathy K. Diabetic retinopathy. StatPearls. 2025. Accessed at: https://www.ncbi.nlm.nih.gov/pubmed/32809640 32809640

[2] Al-Dwairi R, El-Elimat T, Aleshawi A, Vitreous levels of vascular endothelial growth factor and platelet-derived growth factor in patients with proliferative diabetic retinopathy: A clinical correlation. Biomolecules. 2023;13(11). doi:10.3390/biom13111630 PMC10669526 38002312

[3] Cheema AA, Cheema HR. Diabetic macular edema management: A review of anti–vascular endothelial growth factor (VEGF) therapies. Cureus. 2024;16(1):e52676. doi:10.7759/cureus.52676 38264181 PMC10804209

[4] Saint-Geniez M, Maldonado AE, D’Amore PA. VEGF expression and receptor activation in the choroid during development and in the adult. Invest Ophthalmol Vis Sci. 2006;47(7):3135–3142. doi:10.1016/j.cellsig.2007.05.013 16799060

[5] Ford KM, Saint-Geniez M, Walshe T, Zahr A, D’Amore PA. Expression and role of VEGF in the adult retinal pigment epithelium. Invest Ophthalmol Vis Sci. 2011;52(13):9478–9487. doi:10.1167/iovs.11-8353 22058334 PMC3250352

[6] Holmes K, Roberts OL, Thomas AM, Cross MJ. Vascular endothelial growth factor receptor-2: Structure, function, intracellular signalling and therapeutic inhibition. Cell Signal. 2007;19(10):2003–2012. doi:10.1167/iovs.11-8353 17658244

[7] Bontzos G, Bagheri S, Ioanidi L, Nonresponders to ranibizumab anti-VEGF treatment are actually short-term responders: A prospective spectral-domain OCT study. Ophthalmol Retina. 2020;4(12):1138–1145. doi:10.1016/j.oret.2019.11.004 31937473 PMC7416963

[8] Otsuji T, Nagai Y, Sho K, Initial non-responders to ranibizumab in the treatment of age-related macular degeneration (AMD). Clin Ophthalmol. 2013;7:1487–1490. doi:10.2147/OPTH.S46317 23901256 PMC3726592

[9] Krebs I, Glittenberg C, Ansari-Shahrezaei S, Hagen S, Steiner I, Binder S. Nonresponders to treatment with antagonists of vascular endothelial growth factor in age-related macular degeneration. Br J Ophthalmol. 2013;97(11):1443–1446. doi:10.1136/bjophthalmol-2013-303513 23966368

[10] Zuber-Laskawiec K, Kubicka-Trzaska A, Karska-Basta I, Pociej-Marciak W, Romanowska-Dixon B. Non-responsiveness and tachyphylaxis to anti-vascular endothelial growth factor treatment in naïve patients with exudative age-related macular degeneration. J Physiol Pharmacol. 2019;70(5). doi:10.26402/jpp.2019.5.13 32009630

[11] Young TA, Wang H, Munk S, Hammoudi DS, Young DS, Mandelcorn MS, Whiteside CI. Vascular endothelial growth factor expression and secretion by retinal pigment epithelial cells in high glucose and hypoxia is protein kinase C dependent. Exp Eye Res. 2005;80(5):651–662. doi:10.1016/j.exer.2004.11.015 15862172

[12] Ahn YT, Chua MS, Whitlock JP Jr, Rodent-specific hypoxia response elements enhance PAI-1 expression through HIF-1 or HIF-2 in mouse hepatoma cells. Int J Oncol. 2010;37(6):1627–1638. doi:10.3892/ijo_00000817 21042733

[13] Callan A, Heckman J, Tah G, VEGF in diabetic retinopathy and age-related macular degeneration. Int J Mol Sci. 2025;26(11). doi:10.3390/ijms26114992 PMC12154301 40507802

[14] Hazim RA, Volland S, Yen A, Burgess BL, Williams DS. Rapid differentiation of the human RPE cell line, ARPE-19, induced by nicotinamide. Exp Eye Res. 2019;179:18–24. doi:10.1016/j.exer.2018.10.009 30336127 PMC6360117

[15] Cattaneo MG, Chini B, Vicentini LM. Oxytocin stimulates migration and invasion in human endothelial cells. Br J Pharmacol. 2008;153(4):728–736. doi:10.1038/sj.bjp.0707609 18059319 PMC2259201

[16] Cassoni P, Marrocco T, Bussolati B, Oxytocin induces proliferation and migration in immortalized human dermal microvascular endothelial cells and human breast tumor-derived endothelial cells. Mol Cancer Res. 2006;4(6):351–359. doi:10.1158/1541-7786.MCR-06-0024 16778082

[17] Elsayed ASI, Azab AE, Etwebi KA. An overview of oxytocin: Chemical structure, receptors, physiological functions, measurement techniques of oxytocin, and metabolism. J Clin Res Rep. 2022;11(4):1–11. doi:10.31579/2690-1919/256

[18] Zhu J, Wang H, Zhang X, Xie Y. Regulation of angiogenic behaviors by oxytocin receptor through Gli1-induced transcription of HIF-1α in human umbilical vein endothelial cells. Biomed Pharmacother. 2017. doi:10.1016/j.biopha.2017.04.021 28445928

[19] Gauquelin G, Geelen G, Louis F, Presence of vasopressin, oxytocin and neurophysin in the retina of mammals: Effect of light and darkness, comparison with the neuropeptide content of the neurohypophysis and the pineal gland. Peptides. 1983;4(4):509–515. doi:10.1016/0196-9781(83)90056-6 6647119

[20] Elsayed ASI, Azab AE, Etwebi KA. An overview of oxytocin: Chemical structure, receptors, physiological functions, measurement techniques of oxytocin, and metabolism. J Clin Res Rep. 2022;11(4):1–11. doi:10.31579/2690-1919/256

[21] Degirmenci C, Afrashi F, Erbas O, Aktug H, Taskiran D. The preventive effect of oxytocin on retinopathy in streptozotocin-induced diabetic rats. Turk J Ophthalmol. 2019;49(2):68–72. doi:10.4274/tjo.galenos.2018.47897 31055890 PMC6517859

[22] Correa-da-Silva F, Kalsbeek MJ, Gadella FS, Reduction of oxytocin-containing neurons and enhanced glymphatic activity in the hypothalamic paraventricular nucleus of patients with type 2 diabetes mellitus. Acta Neuropathol Commun. 2023. doi:10.1186/s40478-023-01606-w PMC10318717 37400893

[23] Heimsath EG, Unda R, Vidro E, Muniz A, Villazana-Espinoza ET, Tsin A. ARPE-19 cell growth and cell functions in euglycemic culture media. Curr Eye Res. 2006;31(12):1073–1080. doi:10.1080/02713680601052320. 17169846

